# Product News

**DOI:** 10.1155/S1463924683000164

**Published:** 1983


Journal of Automatic Chemistry, Volume 5, Number 1 (January–March 1983), pages 49–62

### Product News

### Instruction-free GC

Packard Instrument Ltd, a division of United Technologies, have announced the sixth gas chromatograph in its microprocessor-based 'Blue Series'. The new machine, Model 439, adds interactive CRT/keyboard programming to the previous units; the programming facility is instruction-free: an index page lists available programs and, when a desired program is called, the operator is guided through it step by step. A 'Help' button is included, which brings a concise explanation of the system (in a choice of several languages) into play.

The CRT has a 9 in screen with brightness control and anti-reflection protection. Image burn-in is avoided by an automatic system which switches off long-standing displays and rolls a reminder message on screen. Parameters being programmed appear in reverse video on the program page, while data are entered in a fixed entry field.

The 439GC has a sliding electronic compartment which allows access to all boards from the front of the instrument. A diagnostics program is included for easy location of board errors and other relevant service information.

The keyboard was designed to be simple. It has direct-acting attenuation and valve-switching controls. As a time saver, there are page programs and individual line programming facilities. The status page displays all operating parameters for both channels, including the particular detector controller which is mounted in each channel.

Other features include permanently stored programs; external events control of 13 relays; storage of six complete GC methods; the possibility for control of four satellite GCs; autoranging electrometer with a linear dynamic range of nine decades; time programming and RS-232 interface capabilities.

The 439 still has the Packard 'analytical module' concept: interchangeable dual injectors/columns/detectors units. A full range of plug-in detectors and injectors is available. There is 360° column access at eye level through the pneumatically raised analytical module; near-ambient operation by a proportionally controlled oven door; a low-power consumption oven with oven heating switch-off facility; and easy connection of capillary columns (stainless-steel, glass or fused silica). Capillary column injectors (splitter, splitless, solid injector) including a new on-column injector are part of the new instrument's design features.

*Further information from Packard Instrument Ltd, 13–17 Church Road, Caversham, Berkshire RG4 7AA, UK. Tel.: 0734 478234.*

**Circle No. 6 on Reader Enquiry Card**

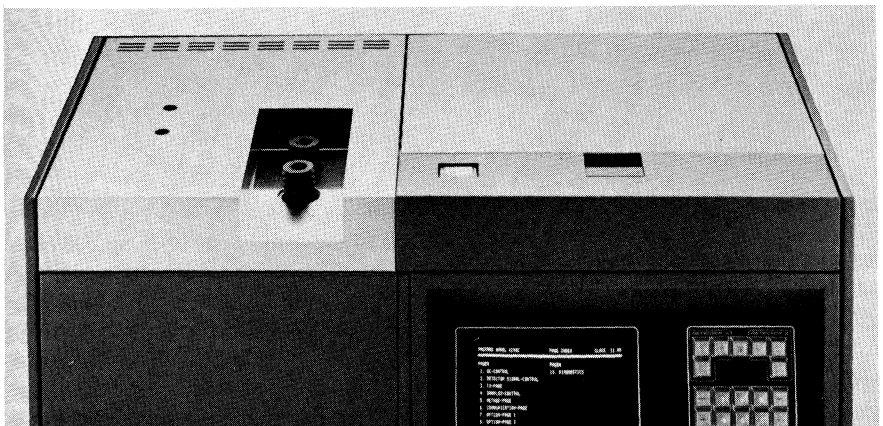

*Model 439: the sixth gas chromatograph in Packard Instrument Ltd's 'Blue Series'. The manufacturer claims that the 439GC has one of the market's best price/performance values. (Packard Instrument Ltd, Caversham, UK.)*

### Sage pumps

A leaflet describing the Sage range of syringe-infusion and peristaltic pumps is offered by Arnold R. Horwell Ltd. The range, manufactured by Orion, includes five models which are designed to provide infusion or withdrawal of liquids; continuously variable or pre-selected flow rates; and multiple syringe capacity. Current applications of the pumps include drug infusion into laboratory animals, feeding samples to atomic absorption spectrophotometers, flame photometers, titrators etc. and sampling in water or air analyses.

*Copies of the leaflet from Arnold R. Horwell Ltd, 2 Grangeway, Kilburn High Road, London NW6 2BP.*

**Circle No. 7 on Reader Enquiry Card**

### Energy-dispersive spectrometer

A fully automated version of the PV 9500 energy-dispersive spectrometer has been launched by Philips. The PV 9500/80 includes a computer-based system for qualitative and quantitative analyses linked to an automated sample-analysis and sample-changing system, which offers sequential analysis of up to 15 samples.

A feature of the PV 9500/80 is a full vacuum/helium flush capability for enhanced light-element detection. Operators have a choice of three sample-changing modes: manual—allowing the selection of a single sample; semi-automatic—for analysis of a sample at the touch of a button; and the fully automatic loading and analysis of up to 15 samples in sequential order.

*The spectrometer will be marketed by Pye Unicam Ltd, from whom further information is available: York Street, Cambridge CB1 2PX, UK. Tel.: 0223 358866.*

**Circle No. 8 on Reader Enquiry Card**

### Automated enzyme immunoassay module

Developed in collaboration with the Wolfson Research Laboratories (Queen Elizabeth Hospital, Birmingham, UK) Kone Instruments' new enzyme immunoassay module will either stand alone or will fully integrate with the Kone CD analyser. The module was designed to automate the assay of methods which require a solid-phase separation step. Specifically, it controls the automation and handling of antibody-coated polystyrene beads—a popular separation phase which is regarded by many workers as preferable to tubes or microtitre plates, and is now used by several manufacturers of EIA kits. Four batches of 24 tubes are processed in different positions in the module, which means that up to 96 sample tubes can be analysed simultaneously. Each bead is suspended in an individual spring-holder in groups of 24 for each of the four matrix plates. When incubation begins, the matrix plate automatically descends into the sample tube containing reagent, and a controlled agitation of the bead effects mixing. After incubation the plate automatically rises to achieve separation of the solid phase. When used in conjunction with the Kone D dispensing unit and the 24-channel Kone C photometric analyser, the module allows a wide range of immunoassays to be mechanized. The module is under microcomputer control and the user can select assay incubation periods and control temperatures through a standard keyboard. A numeric display indicates incubation times and an audible warning signal is given at the end of an incubation. Procedures for several key assays such as AFP, Ferritin and $T_4$ are offered, and a growing number of applications are available from Kone's technical support group.

*Details from Peter Gibson, Kone Instruments, Regent House, Heaton Lane, Stockport, UK. Tel.: 061 477 0662.*

**Circle No. 9 on Reader Enquiry Card**

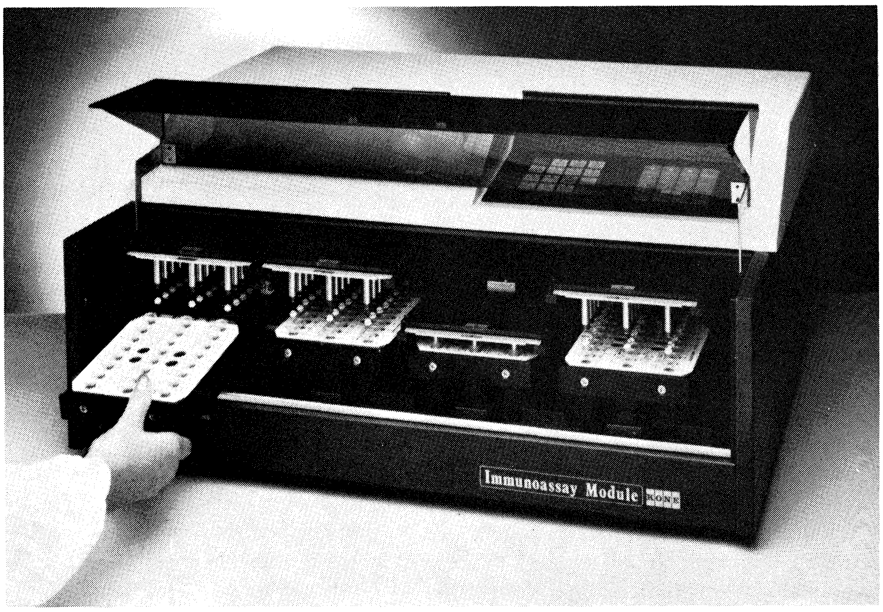

*The new Kone automated enzyme immunoassay module, designed to automate the assay of methods which require the handling of antibody-coated polystyrene beads. (Kone Instruments, Stockport, UK.)*

### Water purification

*How to Control Water Quality for Consistent Test Results* is a brochure from Hydro describing their pre-treatment, treatment, and post-treatment water purification technologies and comparing them for use and cost-effectiveness. A two-page translucent overlay graphically illustrates how the flow pattern through a Hydro water system ensures consistency of water quality. And the brochure further compares the cost of a Hydro custom system with disposable cartridge systems. The company designs reverse osmosis, carbon adsorption, and deionization units, pre- and post-filters, as well as ultra-violet sterilization and ultrafiltration units to suit any specific purity level, volume or throughput need.

*Details from Hydro, PO Box 12197, Research Triangle Park, North Carolina 27709, USA. Tel.: 919 942 8786.*

**Circle No. 10 on Reader Enquiry Card**

### EDT Research/Bruker SA

As a result of corporate restructuring, Bruker SA of Brussels, a subsidiary of Bruker Spectrospin of Karlsruhe, have decided to withdraw from the electrochemical instruments market and have reached an agreement with EDT Research to take over the production and distribution of their full range of instruments. This transfer will mean that EDT will change from the status of importer to that of producer and exporter (since all existing foreign representatives of Bruker SA will continue to act for EDT). The company have already begun production, at Park Royal, of the range of polarographs, potentiostats, cells and accessories for which Bruker have had a good reputation. The products will appear under the EDT name and should be available by January 1983. Until then EDT have sufficient stocks of Bruker-produced instruments to meet foreseeable demand.

*Catalogue*

In order to enable potential users to make a more informed choice from its range of available monochromators, Jobin-Yvon have published a catalogue. It lists, and briefly describes, all of the company's instruments, from the low-cost H10-61 model to the 3 m Ultra High Vacuum Grazing Incidence Spectrometer. The free catalogue could be useful reference for chemists, physicists and engineers working with light. EDT are distributing the brochure in the UK.

*Applications note*

EDT also offer an applications note which describes recent determinations using the Model LCA15 electrochemical detector in HPLC. The note, called LC9, gives brief descriptions of the chromatographic conditions and detection parameters for such drugs as penicillamine, glisoxepide, morphine and paracetamol and phenothiazine. The use of gradient elution with electrochemical detection is discussed and a list of species recently determined with the method is provided.

*Further information, and copies of the leaflets, from EDT Research, 14 Trading Estate Road, London NW10 7LU. Tel.: 01 961 1477.*

**Circle No. 11 on Reader Enquiry Card**

### Chromatography at Laboratory '82

Several new chromatography systems were introduced to the UK at September's Laboratory '82 Exhibition by Laboratory Impex Ltd. These included a new data-processing system for the LB 503 and 504 ranges of high-sensitivity *radio HPLC* detectors. The detectors are widely used in pharmaceutical research (Laboratory Impex recently published a brochure describing applications in the pharmaceutical industry—this is available now free of charge) and the DP addition will speed up data eveluation and plotting of chromatograms.

A DP system for the company's LB 272 *radio thin-layer chromatography* scanner and the Berthold Linear Analyser was also demonstrated at the meeting. The new system is based around a microcomputer and will allow automatic peak integration and expression of peaks as a percentage of total activity. High-quality hard-copy print-outs of chromatograms, with peak integrals and facilities for storing large numbers of runs on floppy disc with fast subsequent recall, are also major features of the new system.

The *Auto-Biolumat LB 950* is a new and fully automated bioluminescence/chemiluminescence analyser, which includes a number of 'patent-applied-for' features. The analyser has been designed on a modular basis for ease of use and maximum flexibility, and comprises a sample-processing stage that can add up to four reagents to the sample, provide automatic sample incubation and specially selected photomultiplier detector head. The data from the sample stage is processed by a 48k microcomputer with floppy disc drive; a VDU displays histograms, activity curves etc.; a printer/plotter provides a hard-copy print-out. The detector head uses a novel light-sealing method, currently the subject of a patent application, to eliminate all ambient light. This feature, combined with the very high sensitivity of the photomultiplier, allows bioluminescent labels to be detected at concentrations as low as $10^{-13}$mol and chemiluminescent labels as low as $10^{-18}$mol. Special precautions have been taken to eliminate the risk of corrosion caused by the highly reactive reagents used in chemiluminescence studies. All surfaces that are liable to come into contact with reagents are Teflon or chrome-nickle steel. When measuring very fast reactions it is important to ensure rapid, even mixing of the reagent and sample, and to initiate the measuring cycle virtually instantly or valuable information may be lost. The LB 950 uses a special reagent-injection method that has proved to be more effective than mechanical stirring. Reagent injection is also made at the detector stage so measurements can commence immediately. The LB 950 uses a serpentine sample chain that can accommodate up to 400 samples at any one time.

*More information from Laboratory Impex Ltd, Lion Road, Twickenham, Middlesex, UK. Tel.: 01 891 4881.*

**Circle No. 12 on Reader Enquiry Card**

### Continuous particle-monitoring system

Remote, multi-point monitoring for particulate contamination in air can be performed continuously, and unattended, with Climet's CI-210 monitoring system. Up to 48 locations and/or sensors may be monitored simultaneously for particle count; and sensors for determining relative humidity, temperature, conductivity, resistivity and air-flow velocity can be added for complete facility monitoring. Airborne particles down to 0·3 μm and liquid-borne particles to 0·5 μm are detected, counted and recorded. Individual alarms for each sensor are operator-settable via a front-panel keyboard. The system could be used to monitor clean rooms, environmentally-controlled areas, laminar flow benches, computer disc drives, pharmaceutical-fill stations and semiconductor manufacturing areas.

*Details from Climet Instruments Company, 1320 W. Colton Avenue, PO Box 151, Redlands, California 92373, USA. Tel.: 714 793 2788.*

**Circle No. 13 on Reader Enquiry Card**

### Microelisa reader

A colour leaflet that outlines the increased versatility, particularly for patient data storage applications, which can be achieved with their Microelisa Mini-Reader when linked to a programmable-HP41 calculator for Elisa routines, is offered by Dynatech Laboratories. The Mini-Reader is basically a compact photometer and can process a 96-well Microelisa plate in approximately 3 min. The light absorbance value of each well is displayed; and it is possible to record data on-line, store it and obtain a print-out in which each of the 96 wells is readily

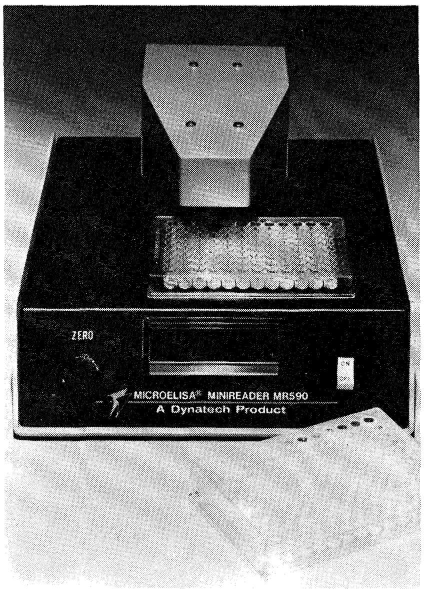

*The Microelisa Mini-Reader. Its features include reading through the wells of a Microelisa plate, reading both flat and 'U' bottom plates, linear measurement from 0 to 15 absorbance units and a precision of ±0·01 absorbance units. The Reader is extremely compact and portable. (Dynatech Laboratories Ltd, UK.)*

identified. The HP41 calculator can be pre-programmed from bar codes so that it transfers results in a logical sequence with automatic well designation.

*Copies from Dynatech Laboratories Ltd, Daux Road, Billingshurst, Surrey RH14 9SJ, UK. Tel.: 040381 3381.*

**Circle No. 14 on Reader Enquiry Card**

### Fraction collector kit

A preparative fraction collection accessory kit is offered for the FRAC-300 fraction collector. The kit contains an electromagnetic valve fitted with ¼ in polypropylene tubing, 10 preparative funnel racks, 50 m of PVC tubing and a trolley. The valve, which is fitted to the delivery arm in place of the normal flow stopper, can be used at flow rates up to 1 l/min and at pressures up to 0·2 MPa. Valve operation can be synchronized with delivery head movements—so it is closed during movement between funnels and racks and after collection of the last fraction. Connection to the chromatography system is made with standard ¼ in connections. Each preparative rack has five funnels enabling the collection of 50 large volume fractions per cycle. The PVC tubing leads from the funnel through a hole in the top shelf to the collection vessels below, which may be placed on the bottom shelf or on the floor.

Two further items are available, both are intended to increase efficiency and precision. One is a contractor box which prevents build-up of pressure within the system; the other a flowmeter cable for interfacing the FRAC-300 with a Bearinglass flowmeter so that precise volume fractions can be collected independently of variations in flow rate.

The preparative kit means that the FRAC-300 system is now equally suitable for the development and optimization of chromatography purification schemes at the laboratory bench and for the checking and running of a scheme on a pilot plant scale.

*Further information from Dr Liz Hill, Pharmacia Fine Chemicals, Prince Regent Road, Hounslow, Middlesex TW3 1NF, UK. Tel.: 01 572 7321.*

**Circle No. 15 on Reader Enquiry Card**

### Quadrupole gas chromatograph/ mass spectrometer

Finnigan MAT's 4600 series, a research-grade quadrupole gas chromatograph/ mass spectrometer, is described in a new data sheet. The 4600 series offers a mass range of 4 to 1800 $\mu$ through the use of hyperbolic rods. The data sheet gives specifications, hardware features and lists the available optional equipment. The system is specifically designed to perform GC/MS analyses on higher molecular weight samples, which are done typically in the fields of energy and biomedical research.

Finnigan MAT has operations in San Jose, California, and Bremen, FR Germany; the company provides a full service product line to the mass spectrometry community, offering instruments for organic and inorganic analyses.

*For more information contact the Marketing Communications Department, Finnigan MAT, 355 River Oaks Parkway, San Jose, California 95134, USA. Tel.: 408 946 4848.*

**Circle No. 16 on Reader Enquiry Card**

### Haematology analysers

The S-Plus III and S-Plus IV haematology analysers both offer a 12-parameter blood profile with a throughput of 115 samples/h. The S-Plus IV has a whole blood sampling volume of 100 $\mu$l so it is particularly useful for paediatric and geriatric applications; it also cuts down reagent consumption by 21% compared with previous S-Plus models.

The S-Plus III retains the S-Plus facility for using pre-diluted blood and can be used in conjunction with the CASH (Coulter Automatic Sample Handler) for the complete automation of sampling and mixing patient blood samples.

A data terminal, the Coulter QC, can be added to both models. The terminal provides a four-element quality-control package and also offers two further blood parameters: Lymph % and Lymph number. The four elements incorporated in the QC data program are automatic daily instrument checks, storage and analysis of control blood data, $\bar{X}_B$ moving average using patient samples and, finally, 'technologist review', allowing a cross-check using the data from the other three elements. Hard copy of all data, including size-referenced histograms for RBC, WBC and platelets, can be produced by the standard printer/plotter with a choice of full- or half-size print-outs.

*Details of the two new S-Plus models from Coulter Electronics Ltd, Northwell Drive, Luton, Bedfordshire LU3 3RH, UK. Tel.: 0582 582442.*

**Circle No. 17 on Reader Enquiry Card**

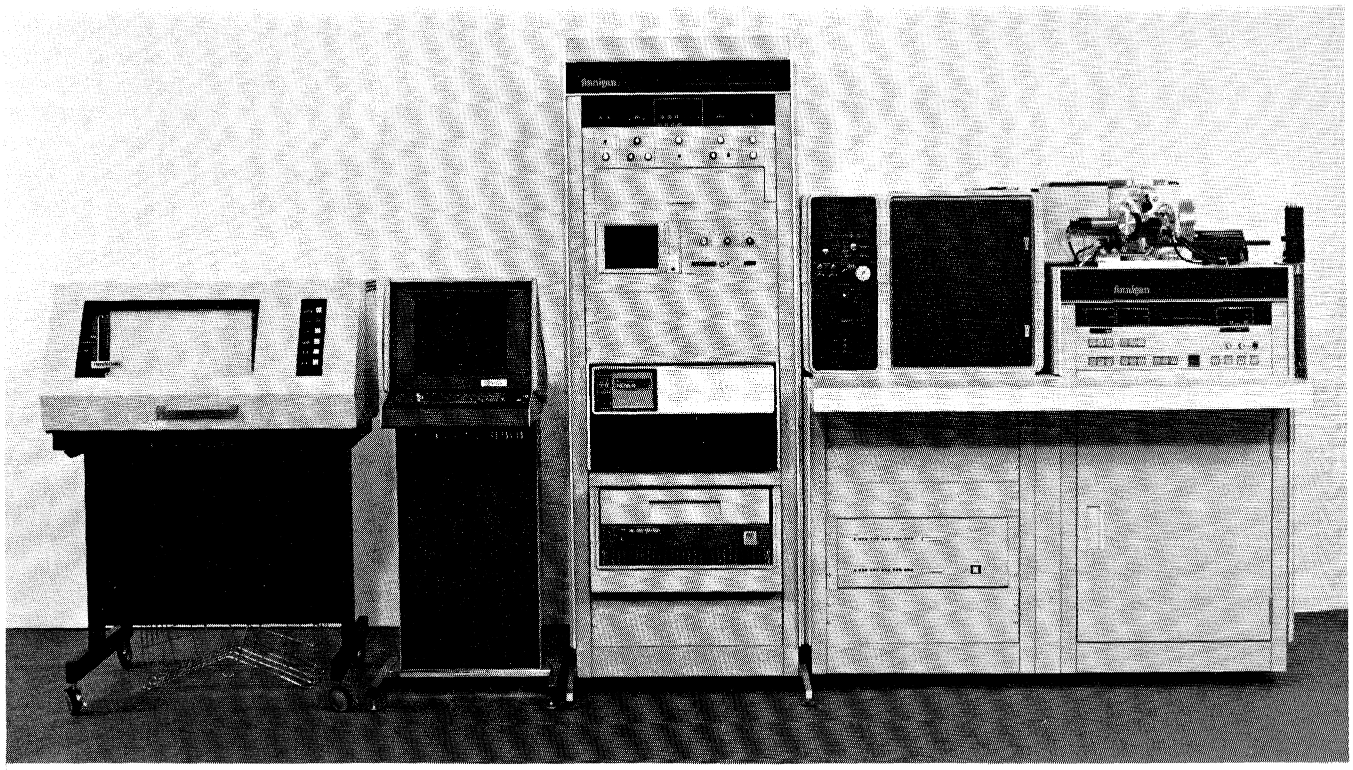

*The 4600 series—a quadrupole GC/MS which is particularly suited to analyses in energy and biomedical research where compounds of high molecular weights are encountered. Sample introduction methods available with the series include gas chromatograph, liquid chromatograph, solids sample probe, and a direct evaporation probe. Several ionization methods are offered with a versatile, interchangeable ion-volume design that permits fast, easy switching of EI and CI volumes for optimum results. (Finnigan MAT, USA and FR Germany.)*

### Furnace for AA spectrophotometry

The SP9 furnace is a new addition to Pye Unicam's range of video furnaces; it shares several of the series' features but has a simplified control system. The SP9 is designed for Pye Unicam's SP190, SP2900 and SP9 atomic absorption spectrophotometers.

The temperature of the furnace's cuvette is controlled via a fibre-optic/silicon photodiode feedback system, calibrated for the life of the unit and requiring no operator adjustment. This gives rapid heating, over 2000°C/s, for 'atomize' stages and also accurate temperature control for high-temperature ash stages. The temperature of the cuvette may be programmed up to 3000°C. Condensation effects in the cuvette, a traditional problem with furnaces, are eliminated by a unique gas flow system. The head also features an automatic door, open for injection but closed during operation to protect the cuvette from contamination and atmospheric oxygen. The cuvette head fits onto the atomic absorption instrument in place of the burner, without the need to remove the spray chamber. This gives rapid change from flame to furnace atomization.

A range of ramp rates may be programmed for ashing complex matrices and a dry/ash programme is included for easy standard-additions operation. Automatic base-line correction may be selected before every atomize peak, if required.

Interlocks are built-in for inert gas, cooling water, door operation, cuvette head temperature and cuvette power-supply.

*Details from Pye Unicam Ltd, York Street, Cambridge CB1 2PX, UK. Tel.: 0223 358866.*

**Circle No. 18 on Reader Enquiry Card**

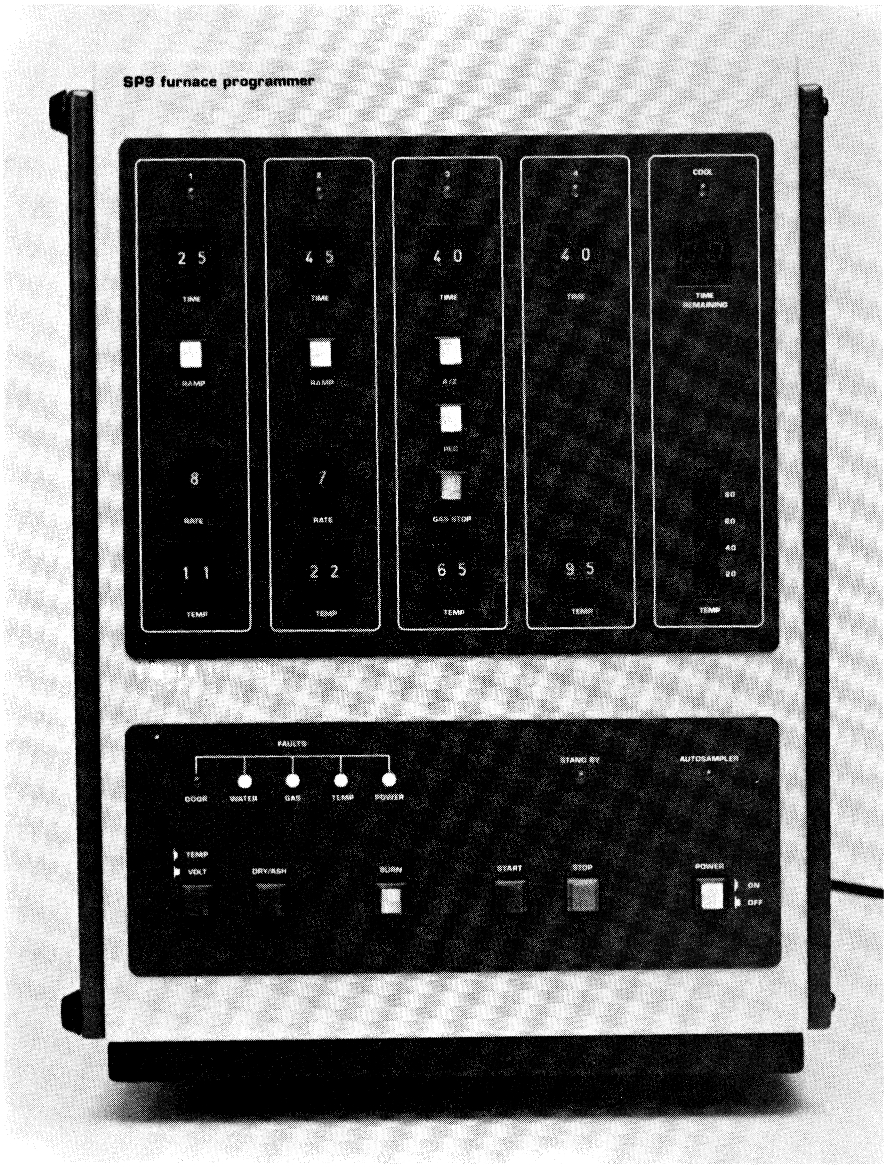

*The programming unit of the SP9 furnance for atomic absorption spectrophotometry. (Pye Unicam Ltd, Cambridge, UK.)*

### Electronic balance

The Fisher/Ainsworth SC-200 is an electronic top-loader balance which has a capacity of 200 g. With ±0·1 g sensitivity and precision, it provides a 2s response time, 0·1 to 49·9 g non-subtractive electronic taring range, and a 1s taring time. Built for field, factory or laboratory use, the SC-200 has a four-digit liquid crystal display (so it features cool-running electronics) and an electronic filter that corrects for environmental variables. The balance is light enough and small enough to be carried in a briefcase. And the SC-200 is cheap to run: it needs a 9V battery and automatically shuts off after 90 s to conserve battery life. An optional 115 V a.c. adaptor for continuous operation is also available.

*A bulletin describing the SC-200 is available from Fisher Scientific Company, 711 Forbes Avenue, Pittsburgh, Pennsylvania 15219, USA.*

**Circle No. 19 on Reader Enquiry Card**

### Analyzer II

An analyser which, it is claimed, exceeds the expected capabilities of a bench-top chemistry system, which has been designed to perform with high precision and which has features that are generally associated with instuments far above its price range, was announced at the end of October 1982. Worthington Diagnostic Systems' 'Analyzer II' is controlled through a simple keypad; it is capable of integrating up to five standards; and end-point, kinetic and immunoassays are automatically calibrated and results stored for later use. Test parameters can be modified before or during a run; in addition, a data-plot mode allows the user to program the analyser for method development and research applications. It is the first bench-top chemistry system able to run patient profiles. The system can be programmed to perform batches of different chemistries to run sequentially and unattended. Routine analysis can be interrupted for 'stat' samples without losing test results; the analyser will automatically continue with routine work after a 'stat' is completed. Sample ID numbers and test requisitions can be entered via the keyboard. The system will produce work-lists and collate results for 60 samples with 15 requisitions each, or more samples with fewer tests. An auto-pipettor follows work-lists by selecting only those samples needed for each chemistry, thus reducing operator time and reagent wastage. The manufacturer produces a full range of matched chemistries for use with the analyser, but almost any reagents are suitable.

*Details from Worthington Diagnostic Systems, Dolphin House, Rockingham Road, Uxbridge, Middlesex UB8 2UE, UK. Tel.: 0895 38451.*

**Circle No. 20 on Reader Enquiry Card**

### Thermometer simulator

The R50 is a resistance thermometer simulator, a purpose-built, high-resolution decade box which has been designed to assist in the calibration of resistance-thermometer measuring devices. The instrument, which costs £145, has an accuracy of 0·05% and any value of resistance may be dialled up, from 0–1111·1Ω in increments of 0·01Ω. Special features include low-temperature coefficient resistors and good repeatability which is guaranteed by the use of self-wiping, maintenance-free switches, each of which has eight parallel connected contacts to reduce contact resistance.

*The R50 is being sold in the UK by J. J. Lloyd Instruments Ltd, 1 Brook Avenue, Warsash, Southampton, Hampshire SO3 6HP, UK. Tel.: 04895 4221.*

**Circle No. 21 on Reader Enquiry Card**

### Soil gas probe

British Gas's soil gas probe, designed at the Corporation's London Research Station, is to be manufactured under licence and marketed by Research Engineers Ltd.

Changes in soil atmosphere often occur as a result of flooding, compaction or pollution: the oxygen concentrations usually decrease markedly, carbon dioxide levels increase, and other gases or volatile compounds (such as hydrogen sulphide or hydrocarbons) may be formed or introduced. So a probe which can measure soil atmospheres is likely to be of interest to local governments and others who wish to detect or measure concentrations of methane generated in refuse tips; waste-tip operators will also find it useful to detect hydrogen sulphide and other volatile compounds. And it is important to horticulturalists, foresters and farmers for identifying oxygen deficiency, which results in poor plant growth. The probe can also be used to highlight waterlogged conditions or compaction, to measure the depth of the water table, and to help define soil types and soil porosity. Other applications could include detection of combustible gases from coal-mine seepages, location of underground fires, and investigation of soil pollution. The instrument is simple to use and enables gas samples to be extracted up to a depth of 1 m with minimal disturbance of the soil. This means that a series of readings can be recorded at the same place over a period. Concentrations of gases in the soil can be easily mapped in both horizontal and vertical planes, and, using the necessary analytical techniques, a number of gases can be analysed on the spot.

The probe has been designed to be practical. It is driven into the ground for sampling by using a hammer assembly; by sliding the outer casing of the probe handle up and down, the hammer action of the handle striking an anvil at the top of the rod forces it into the soil. Markings on the side of the rod at 200 mm intervals indicate the depth to which the probe has been driven. Gas samples are drawn off through a side arm on the probe into appropriate analytical equipment or into suitable containers (for laboratory analysis). The outer tube of the probe, the tip, the centre rod and the anvil are made of stainless steel. At the top of the probe, the tubular handle section is made of mild steel, with a guard near the lower end. It is protected with high-density polyethylene to provide electrical insulation up to 22 kV. The overall length of the probe is 1·5 m and the complete assembly weighs 1·8 kg.

*Sales enquiries to Research Engineers Ltd, Orsman Road, Shoreditch, London N1 5RD. Tel.: 01 739 7811.*

**Circle No. 22 on Reader Enquiry Card**

### 'Laboratory Equipment Directory 1982'

Morgan-Grampian have announced the publication of the 1982 edition of the *Laboratory Equipment Directory*. The book is 250 pages of products and services provided by about 1500 British companies. It gives addresses, telephone numbers and telexes; a buyer's guide: listings of companies under more than 3000 products and services; details of trade names; information on foreign distributors of UK products; a guide to associations, societies and institutions serving the laboratory equipment industry; and a chronological diary of the year's events.

*Cash-with-order price is £13·50, otherwise the 'Directory' costs £15·00. The 1983 volume will be available in May/June next year. Orders to Morgan-Grampian Book Publishing Co. Ltd, 30 Calderwood Street, London SE18 6QH.*

**Circle No. 23 on Reader Enquiry Card**

### Anachem, Gilson and LC

Gilson International's HPLC products—a gradient system, a data-handling package, a fluorescence monitor and a sample-preparation unit—are being sold in the UK by Anachem. The major theme of the product range is the development of software and interfaces for the Apple microcomputer, which gives a flexible, easily updated control unit. The gradient system is compact and upgradeable and is specified for all separations between analytical and large-scale preparative LC.

The data master package allows simultaneous dual-channel recording, followed by production of re-scaled chromatograms and fully quantified results. Raw data and reports may be stored on discette for subsequent recall and replay. The package is available either fully integrated into the gradient system software or as a separate unit.

The 212 XYZ liquid handler is a new approach to the problem of sample preparation prior to HPLC analysis. Totally programmable, the 212 is capable of extensive manipulation of samples and diluents in a wide range of tubes and vessels. At the end of sample preparation the 212 acts as an auto-injector to the HPLC system.

The Model 121 fluorometer is a sensitive fluorescence detector for HPLC. Featuring new optical design and circuitry, the 121 can be used for monitoring derivatives or compounds which exhibit native fluorescence. The 121 has cells for both analytical HPLC (9 $\mu l$) and micro-bore LC (0·6 $\mu l$), with selectable time constants to optimize performance.

*Details from (UK) Anachem Ltd, 15 Power Court, Luton, Bedfordshire LU1 3JJ, tel.: 0582 35252; and (USA) Gilson Medical Electronics Inc., Box 27, 3000 W. Beltline, Middleton, Wisconsin 53562, tel.: 608 836 1551.*

**Circle No. 24 on Reader Enquiry Card**

### New distilling unit

After eight years' production the Tecator Kjeltec-II distilling unit 1003 has been replaced by the Model 1028. The 1028 is a twin-distillation unit—there are two independent systems for the automatic addition of reagent to a sample, followed by rapid steam distillation. The Kjeltec 1028 is built around a microprocessor which controls all timing functions; and the instrument incorporates facilities for both macro and semi-micro Kjeldahl determination, as well as several options previously offered as accessories. In addition to Kjeldahl analyses, the Kjeltec 1028 can be used for many other methods, including nitrate in fertilizers and sulphur dioxide in foods and beverages. For low-level work, the 1028 allows the user to ensure freedom from interferences by avoiding the use of tap-water for steam generation. For routine Kjeldahl work, analyses can be carried out at a rate of 36 samples/h.

*Details from Tecator Ltd, Cooper Road, Thornbury, Bristol BS12 2UP, UK. Tel.: 0454 417798.*

**Circle No. 25 on Reader Enquiry Card**

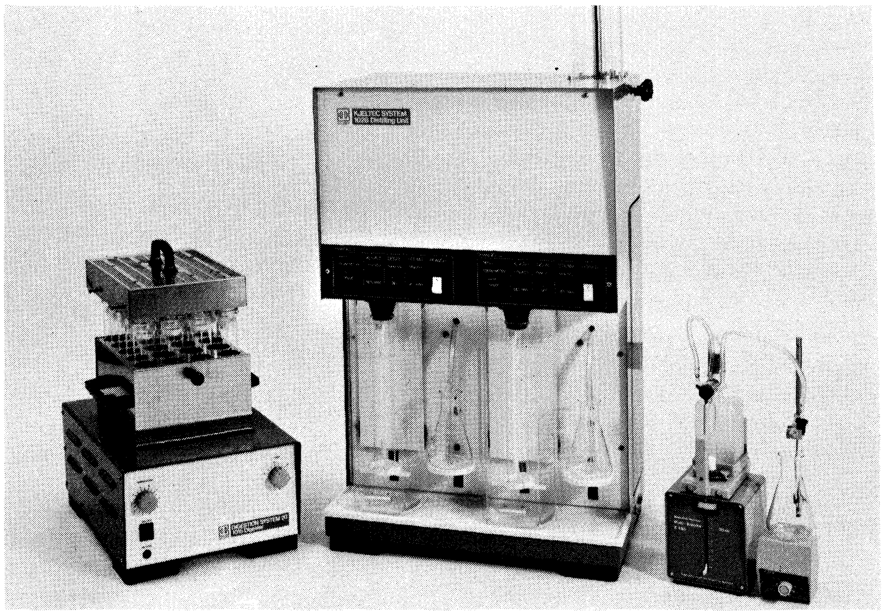

*The Kjeltec 1028 which supersedes the Tecator Kjeltec-II distilling unit 1003. For Kjeldahl and other analyses. (Tecator Ltd, Bristol, UK.)*

### Computer control for Microlab M

A Commodore Pet has been added to the Hamilton Microlab M to create a very flexible diluting/dispensing system. Up to 10 Microlab M pipetting stations can be controlled by one PET: the 10 units can be used individually or interlinked; and all parameters, for each unit, such as speed, volume and mode of operation are monitored and set by the computer. Obviously, as with all computer-controlled systems, the final programs may be very much tailored to each application, by the user. The Microlab M/PET combination is available in two basic formats. For users who already have their own PET there is a package containing one pipetting station, the software and all required accessories. The other system includes the pipetting station, computer, dual disc drive, interfaces and software.

*Details from V. A. Howe & Company Ltd, 12–14 St. Ann's Crescent, London SW18 2LS. Tel.: 01 874 0422.*

**Circle No. 26 on Reader Enquiry Card**

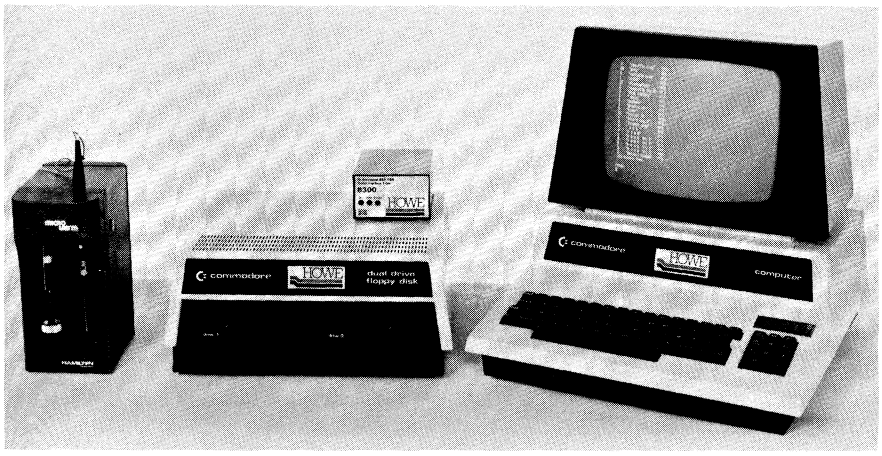

*The Hamilton Microlab M system (one of Hamilton's range of diluter/dispensers) under Commodore PET control. (V. A. Howe & Company Ltd, London.)*

### Portable gas alarm for harsh environments

The need for the anti-corrosion 76GA became evident, according to its manufacturer: Crowcon, from maintenance records. Gas alarms which should last for 10 years or more if regularly serviced were found to need attention after three months when they were used offshore. The alarm for highly corrosive and harsh environments is a specially protected Crowcon 76GA—an established instrument. It is designed to fill the gap between sophisticated, permanently installed protection systems and portable 'sniffers' for spot checks and it provides continuous sampling over a 12 h period. An overnight charge from its built-in mains-powered charger restores the instrument for another full working period. An aspirator unit is provided so that the 76GA can also be used for spot checking, especially in manholes, tanks, ducts or other inaccessible areas. It flashes a red lamp and sounds an audible alarm whenever any gas or vapour—from acetone to xylene—exceeds a pre-set level below the lower explosive limit (LEL). The protection used to combat corrosive conditions includes the use of stainless-steel for the detector block, case, chassis and labels and an epoxy paint finish.

*Further information from Crowcon Instruments Ltd, Temple Road, Cowley, Oxford OX4 2EL, UK. Tel.: 0865 776707.*

**Circle No. 27 on Reader Enquiry Card**

### New syringe dilutors

Four digital syringe dilutors, which together should cover every pipetting/diluting and reagent-dispensing need, have been launched by Dynatech Laboratories. Each model has a microprocessor-controlled stepping motor drive with a separate synchronous motor to power the valves. For rapid interchangeability with other sizes, as well as for ease of cleaning, the syringes are made of glass and Teflon. Each one is supplied with a handprobe incorporating a 'ready-to-deliver' visual indicator. All models have an accuracy of $\geqslant 99\%$ and a precision of $\pm 1\%$ at total syringe volume.

The most versatile model is the DSD 110—this is a pipettor/dilutor/dispenser plus a separate reagent dispenser integrated into one package. The equipment incorporates two separate syringe, valve and delivery tubing arrangements. The set attached to the pipettor/dilutor can be configured to perform a variety of combinations of pipetting/diluting or dispensing over a wide range of volumes. The other set can be programmed to dispense reagents separately or in combination with the dilutor syringe. So the DSD 110 can carry out quite complex fluid-handling tasks. The operator can use the integral membrane-type keypad to program either one or two samples plus reagent, followed by a second independent reagent in one delivery sequence. It is recommended for RIA, EIA or Syva applications, as well as for HCG or TSH applications.

The general-purpose DSD 109 and dual-syringe DSD 108 are similar in appearance to the DSD 110, they have the same keypad design and feature a multi-program memory plus battery back-up. On all three keyboard units, syringe volume and user-selected syringe rates are programmed into the memory. Procedures are then entered directly in microlitres. The DSD 109 is a single syringe unit designed for dilution ratios of 200 : 1 or less. The syringe can be programmed for either one or two samples, with air gap (for RIA, Syva and EIA applications) followed by reagent delivery. With the DSD 108, the user can extend applications to dilution ratios of 200 : 1 and beyond. One syringe is used for sampling, the other for reagent dispensing. The sampling syringe can be programmed to pick up one or two samples with air gap for RIA, EIA or Syva applications. After aspiration, the second syringe dispenses a programmed amount of reagent into the reaction vessel.

The rather simpler and, therefore, cheaper DSD 100 is a general-purpose laboratory pipettor. Thumbwheels are used to control both total and sample volume. A three-way valve enables a variety of combinations of pipetting, diluting and dispensing to be carried out over a wide range of volumes.

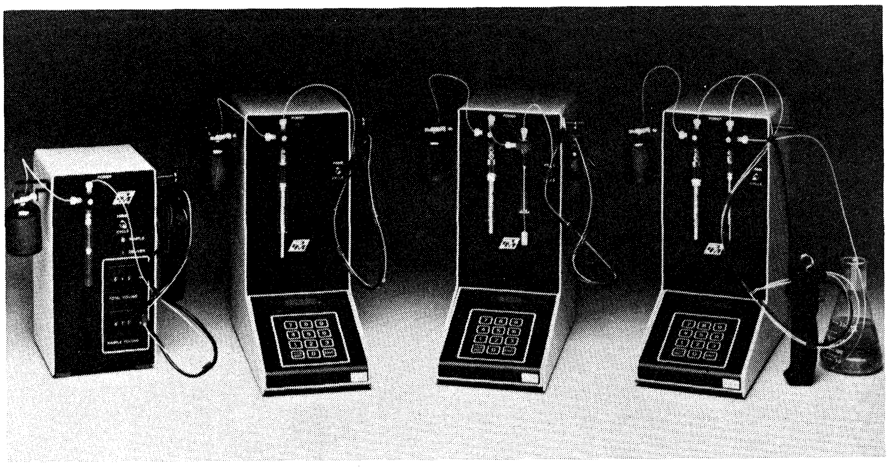

*The range of digital, programmable syringe dilution systems from Dynatech Laboratories, Billingshurst. The four models are intended to cover every pipetting/diluting and reagent-dispensing requirement.*

*Further information from Dynatech Laboratories Ltd, Daux Road, Billingshurst, Sussex RH14 9SJ, UK. Tel.: 040381 3381.*

**Circle No. 28 on Reader Enquiry Card**

### Bio-Rad chart recorder

A chart recorder has been added by Bio-Rad to their range of chromatography equipment; it is available as either a single- or double-pen unit. The decadic gradation of the full-scale values with intermediate ranges allows recording of all values from 1 mV to 100 V over the full scale length. The chart drive varies from 3 cm/h to 1 cm/s and has the facility for reversing the paper flow for convenient location of pen position. The recorder's d.c. servo-system controls operation through a series of integrated circuits; a cut-out and a current-limiting switch in the final stage protects the recorder from overload damage.

*Contact David Forrester, Chemical Division Manager, Bio-Rad Laboratories Ltd, Caxton Way, Holywell Industrial Estate, Watford, Hertfordshire WD1 8RP, UK for further information. Tel.: 0923 40322.*

**Circle No. 29 on Reader Enquiry Card**

### Stack analysis

Columbia Scientific Industries Corporation's Model DS210 stack analysis system is to be distributed in the UK by Techmation. The DS210 system is a novel approach to chemical analysis of stack gases using proven, EPA-certified analysers; it is an integrated method of sample handling, transport and measurement. A precision sampling device prepares the sample, within the stack, at stack temperatures of up to 600°C. The sample is then transported through an unheated sample line to the analysing equipment, without condensation—even at subzero temperatures. The analysis is made with ambient air monitors having high-precision capabilities. Interferences from particulates and other stack gas components have been virtually eliminated.

Ranges from less than 100 ppm to 10 000 ppm full-scale are readily measured with simple field changes, and calibration is performed with high-concentration SRM gases through the sample probe. Multiple stack monitoring is possible with the addition of an extraction module for each additional port.

Most gases for which environmental monitoring might be required can be analysed with this technique: for example $SO_2$, $H_2S$, total sulphur, NO, $NO_x$, $CO_2$ and hydrocarbons; and the DS210 can be used in mobile, as well as permanent, installations.

*For further information on the DS210 stack analysis system contact Techmation Ltd, 58 Edgware Way, Edgware, Middlesex HA8 8JP, UK. Tel.: 01 958 3111.*

**Circle No. 30 on Reader Enquiry Card**

### IBM chemistry

IBM Instruments Inc. have announced two new systems—a bench-top computer system for laboratories and a system for automated liquid chromatography.

### *Laboratory computer system*

Using an IBM 68000 microprocessor and real-time operating software the new system provides instrument control, data acquisition and data analysis for multiple instruments. The system has a modular design so it can be tailored for individual requirements and used as a stand-alone unit, as an instrument controller, or as part of an automated, integrated laboratory system. The system offers printing and plotting capability, data storage on discette and hard disc, large amounts of random access memory, and attachment to instruments and other devices either through standard RS232C and IEEE-488 interfaces or through an optional sensor input/output interface.

Up to four megabytes of on-line storage are available on optional $5\frac{1}{4}$ in and 8 in discettes; $5\frac{1}{4}$ in hard discs can hold up to 40 megabytes. While 128 000 bytes of random access memory are standard in the system, additional memory is also available on feature cards holding up to 1 megabyte each, up to a total of 5 megabytes. The system also provides up to 128 000 bytes of read-only memory.

A 12 in graphics display presents information in either real-time or post-run mode; the screen can display up to 30 lines of text, 80 characters per line, and graphics with a resolution of 768 by 480 dots. Display software also permits 'windowing', so that different sections of the screen can display data from several instruments concurrently.

A high-resolution, colour printer/plotter produces quality printing on plain paper. More than one colour may be used to plot curves, chromatograms or spectra to make interpretation of data easier.

IBM Instruments provide assembly language support and instruction in BASIC. The company will offer Fortran 77 and Pascal compilers, as well as full screen editing, a program library of mathematical and statistical subroutines, and communications support which will permit the system to communicate with other computers.

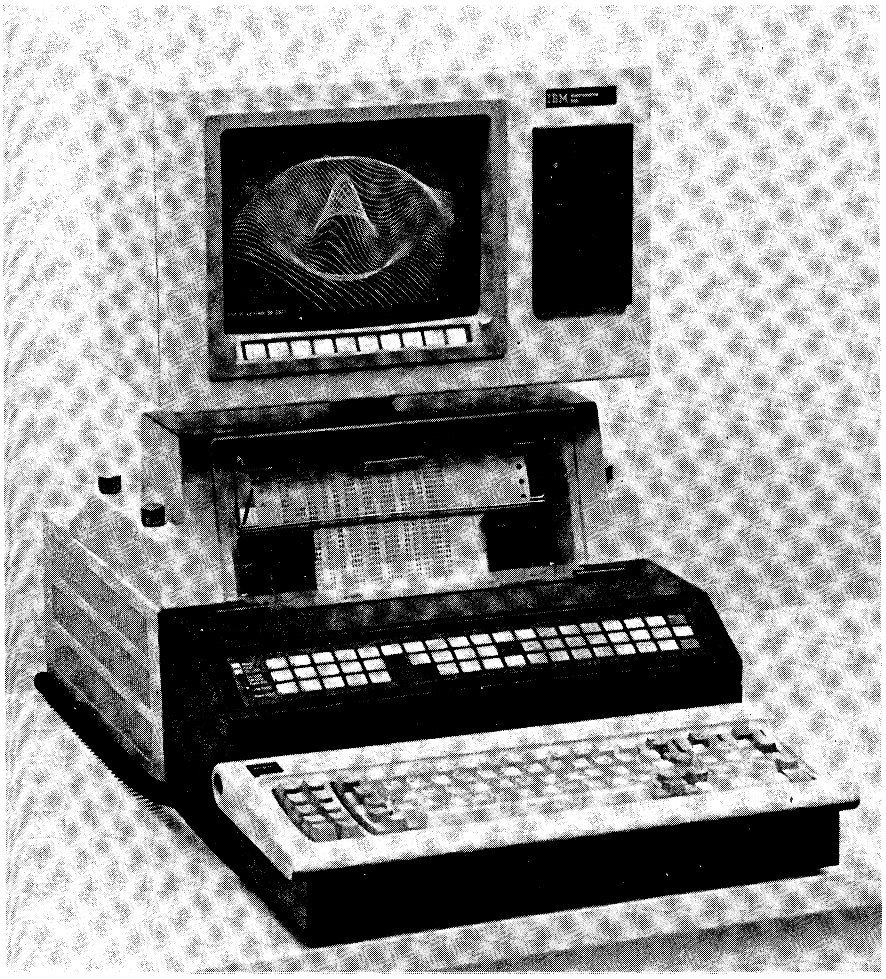

*The IBM Instruments computer system for laboratories, which provides instrument control, data acquisition and data analysis. Results can be presented on a CRT display or in hard copy and can be communicated to other systems.*

### *LC/934 Automated Liquid Chromatography System*

IBM Instruments Inc.'s liquid chromatography system combines a chromatograph and a computer with high-resolution graphics. The chromatograph has a pulse-free pump, ternary gradient capability and a variety of sensitive detectors. Co-ordination of sample data and results permits unattended operation with an automatic sampler. The computer system features a keypad for control of the chromatograph and application software. With the chromatography applications program, up to four IBM liquid chromatographs can be controlled. Other makes of liquid or gas chromatographs can be controlled with user-written software. Data can be acquired and analysed from up to four detectors.

A high-resolution 12 in CRT displays multiple chromatograms at the time data is being collected, or post-run from stored data points; the CRT can also display the current status of any channel or procedure and the listing of programs and calculated results. Ten user-programmable keys on the CRT permit interaction with the CRT for data manipulation.

An optional printer/plotter gives coloured reports on plain paper. Plots are fully annotated, including such items as retention times, peak names, integration start/stop marks, external event actuations and attenuation changes.

The system also performs a wide range of data calculations, such as area percentage, normalization, internal and external standards, single or multi-level and linear or non-linear calibrations.

Programs, data points, and results can be stored on both $5\frac{1}{4}$ in or 8 in floppy discettes. Programs can be created in BASIC for customized reporting and special applications. The system is expandable to performing other computer functions in the analytical laboratory.

*Details from IBM Instruments Inc., Orchard Park, Post Office Box 332, Danbury, Connecticut 06810, USA. Tel.: 203 796 2453.*

**Circle No. 31 on Reader Enquiry Card**

### Sampler for Microlyte

An intelligent automatic sampler for use with the Microlyte ion-selective analyser was introduced by Kone at the end of 1982. Samples from the 30-place rotor are presented at the rate of one/min and the sampler communicates directly with the Microlyte.

Kone's Microlyte measures sodium, potassium and calcium ions ($Na^+$/$K^+$/$Ca^{++}$) using ion-selective electrodes which require no maintenance. A microcomputer has meant a reduction in the mechanics and fluidics in this type of instrumentation and many of the functions have been replaced by software. The result is a reliable ion-selective analyser at a lower cost, and with added features such as a comprehensive self-checking facility and integral on-line interface. The microcomputer-controlled operation includes fully automatic calibration and sampling. An integral printer gives, after 60s, a sample identification, and information on sample type, date and time, as well as a simultaneous print-out of sodium, potassium and calcium results.

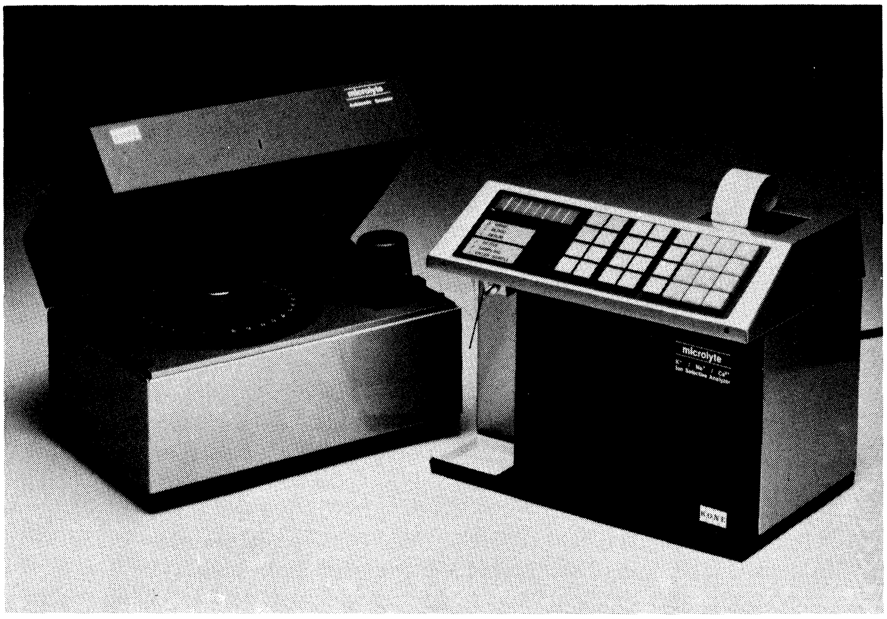

*The Microlyte ion-selective analyser and its new sampler. Whole blood, serum, plasma or prediluted urine can be analysed and, in addition to automatic sample introduction, small (50 or 100 μl samples) can be presented in capillary tubes. (Kone Instruments, UK.)*

*Enquiries to Peter Gibson, Kone Instruments, Regent House, Heaton Lane, Stockport, Cheshire SK4 1BS, UK. Tel.: 061 477 0662.*

**Circle No. 32 on Reader Enquiry Card**

### Electrolyte instrumentation brochure

The System E4A—a new electrolyte analyser which can perform four electrolyte estimations (Na, K, Cl and $CO_2$) simultaneously—is discussed in a brochure written by Beckman-RIIC Ltd. The brochure provides useful information for the laboratory requiring a set of flame-equivalent answers for sodium and potassium determinations without the need for a flame technique and its attendant problems. In addition to the advantage of using a 'flameless' technique, the E4A has the ability to calculate the anion gap for each electrolyte panel, processing over 100 samples/h and without a warm-up time.

Over 400 electrolyte answers can be produced in about 60 min and, with the anion gap calculation for each panel, more than 500 results can be printed out each hour. 'Stat interrupt' and 'manual' facilities allow immediate analysis of stat samples at any time. 50 μl of sample is required. The E4A is built on a modular design, allowing simple replacement of components by the user; plug-in modules are available in kit form.

The brochure also includes details of the system's multi-function computerized keypad and automatic quality-control features.

*The brochure is available from Beckman-RIIC Ltd, Progress Road, Sands Industrial Estate, High Wycombe, Buckinghamshire, UK. Tel.: 0494 41181.*

**Circle No. 33 on Reader Enquiry Card**

### Upgradeable haematology analyser

A modular haematology system which can be upgraded as necessary has recently been launched in the UK by Laboratory Impex Ltd. The CC6 is based on a module for counting red and white blood cells which was designed for minimum routine maintenance. The RBC/WBC module needs a sample of 50 μl of whole blood; the sample is diluted and results are available in 8 s. A repeat count mode, which is particularly useful for abnormal counts—it provides an immediate check without further action by the operator, is built in.

Results are displayed on a digital read-out; a printer is also available. The sample diluent fluid and cleaning fluid are supplied in bulk containers connected directly to the instrument; waste can be conveniently piped away. The low sample volume requirement makes the CC6 ideal for use with critical care, neonatal or paediatric patients.

*Expansion*

The most likely primary expansion of any haematology analyser is to provide an additional haemoglobin measuring capability. A dedicated system expansion module to provide this facility sits on top of the basic unit and the enlarged system operates as an integrated unit. The sample does not have to be transferred to the haemoglobin module once the initial counts have been made. So either RBC/WBC or Hgb measurements can be made separately. After each haemoglobin determination the flow cell is flushed with diluent to reduce sample carry-over and automatically zeroed: this ensures that the CV is always less than 3%.

An HCT/MCV module is also available (so the CC6 becomes a five-parameter system); it connects directly to the main instrument and determinations are made simultaneously with the RBC/WBC counts. Other modules are a platelet unit and a blood cell monitor, which follows the passage of blood cells through the instrument detector aperture.

*Full details from Laboratory Impex Ltd, Lion Road, Twickenham, Middlesex, UK. Tel.: 01 891 4881.*

**Circle No. 34 on Reader Enquiry Card**

### Electrodes

Three combination electrodes with a calomel internal element and potassium chloride electrode eliminate the difficulties common with the more typical silver/silver chloride internals—including excessive clogging of the junction and reaction of the electrolyte with some samples. Fisher's new electrodes also offer simplified maintenance and enhanced performance over a wider range of sample types, compared to Ag/AgCl combination electrodes. Since there is no silver ion present to precipitate or react, they can be used with Tris buffers and silver-sensitive samples.

The three styles are intended to provide the right combination for virtually all applications: the standard-size glass body combination suits the majority of routine pH and titration measurements; the micro-size model offers an extra-long (18 cm) body with a $\frac{1}{4}$ in tip diameter for testing in long and narrow vessels; and the standard size, polymer body combination, with bulb protector, is robust enough for field applications and classroom laboratories.

*For additional specifications and information, contact the Fisher Scientific Company and ask for Bulletin No. 647A: 711 Forbes Avenue, Pittsburgh, Pennsylvania 15219, USA. Tel.: 412 562 8546.*

**Circle No. 35 on Reader Enquiry Card**

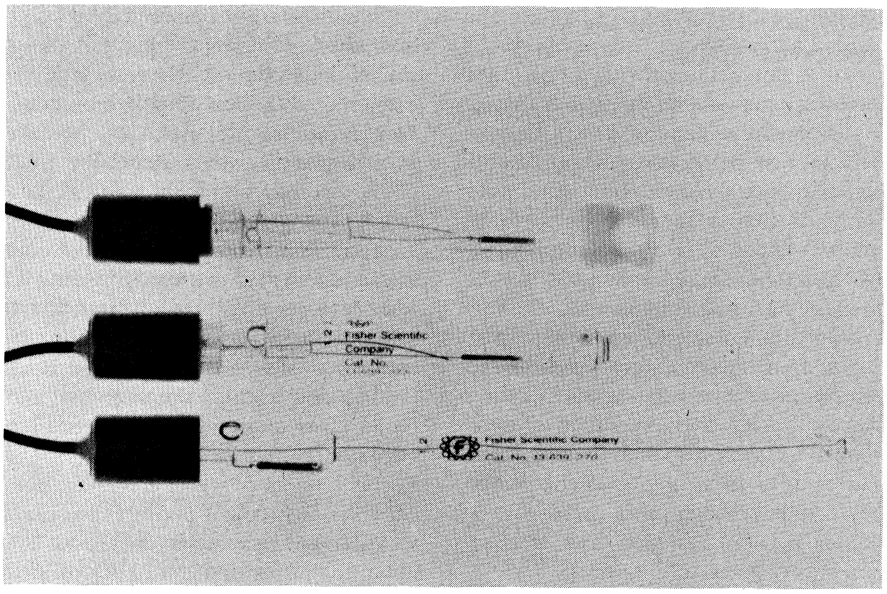

*Fisher's three combination electrodes with calomel references.*

### Chromatographic data acquisition

Based on an Apple II microcomputer, LabLogic's new chromatographic data acquisition system counts all events (digitized input from a U-V detector and/or radio events) during a freely selectable measuring time. The system collects results from up to 1024 measuring periods. A cursor can be moved, even during measurement, to any part of the chromatogram and its position and content will be displayed. Manipulations include integration; selecting time block, sensitivity and level of smoothing; background subtraction; and percentage fraction. For preparative work the program will detect a peak, and a fraction collector can be controlled (variable delay). The system can automatically inject samples one after another. The program organizes the storage of up to 50 chromatograms on disc and a short form print-out before restarting.

*Details from LabLogic, Hallamshire Technical Services, 72 Eldon Street, Wellington Street Industrial Estate, Sheffield S1 4GT, UK. Tel.: 0742 755085.*

**Circle No. 36 on Reader Enquiry Card**

### Spectrometer options

A range of accessories has been added to Pye Unicam's SP7-500 UV/visible spectrophotometer. The extras include an *expansion board*, which introduces the ability to obtain repeat scans, wavelength programming and kinetic measurements. Repeat scanning can be carried out over any selected wavelength sector. It allows the manipulation of spectral data, such as superimposition of spectra with selected absorbance offset between scans, or with unit wavelength displacement—or a combination of absorbance and wavelength offsets, giving a close approximation to three-dimensional presentation. Wavelength programming permits single absorbance measurement and print-out of results at up to 12 pre-selected wavelengths. The kinetic program enables rates to be monitored with user-selectable delay and measurement times.

A microprocessor-controlled *thermoelectric flowcell* is also available, it is used in conjunction with the expansion board and gives precision temperature control to ±0·3°C at five selectable levels. The flowcell provides continuous sample temperature display and enables end-point results to be presented in %T, %A and concentration units, and kinetic measurements to be presented in A/T and rate units.

The option of water-circulating *thermal blocks* offers a low-cost alternative to the thermoelectric flowcell for temperature control of standard 10 mm cuvettes; the requirement for a variety of expensive water-jacketed cuvettes is also eliminated.

An RS232C *computer interface* provides two-way communication for external control and for the storage and manipulation of results, both for the instrument and its accessories.

And two special cell holders further extend the scope of the SP7-500. A *multi-purpose cell holder* allows the analyst to use micro, semi-micro, flow-through and cylindrical cuvettes, as well as the more common rectangular cells. A *long-pathlength cell holder* accommodates conventional cylindrical cells of 22 mm diameter up to 100 mm long. In the case of both of these alternative cell holders, installation is simply a matter of replacing the standard SP7-500 cell turret.

*Information from Pye Unicam Ltd, York Street, Cambridge CB1 2PX, UK. Tel.: 0223 358866.*

**Circle No. 37 on Reader Enquiry Card**

### 50th issue

Issue No. 50—the Jubilee number—of the *CAMAG Bibliography Service* was published in November 1982. The first *CBS* was issued in May 1965; the present number celebrates CAMAG AG's contribution to thin layer chromatography over the years and the increasing popularity of the technique. The *CAMAG Bibliography Service* is an abstracting journal and was described in *JAC*, Vol. 4, No. 4, p. 210.

*Copies from Ch. Gfeller, CAMAG AG, Sonnenmattstrasse 11, CH 4132 Muttenz, Switzerland. Tel.: 061 613434.*

**Circle No. 38 on Reader Enquiry Card**

### Fully automatic hydride generation system

To complement their recently introduced Hydride Generation System, Plasma-Therm Ltd have launched an Autosampler. Specifically designed to take the sample volumes required for hydride analysis and for major element analysis by replicate analysis, the sampler holds 20 samples each with a volume of 50 ml.

Once initiated, the sample probe moves from the wash position into the sample container for the sampling period, determined by either a Plasma-Therm Hydride System or a remote computer. The sampler has a positive drive to the next sample position, and a variable wash period between samples. The sampler also checks that a tube is present prior to sampling and will automatically sequence to the end-of-run position. A repeat sequence of analysis can be initiated externally under software control.

When coupled to the Plasma-Therm Hydride System, the Autosampler allows unattended operation to provide rapid analysis of hydride-forming elements either simultaneously using an ICP system, or sequentially using environmental atomic absorption or furnace techniques.

The instrument has been specifically designed to facilitate the analysis of As, Bi, Sn, Sb, Se, Te, Ge, Hg and Pb in the subparts per billion level. Continuous-flow principles are used, with specific attention being given to stable flow-rates of the sodium borohydride blank and sample streams so that stable conditions are attained in the plasma. The gas/liquid interface vessel allows the hydrogen and hydrides formed to be rapidly transferred into the plasma gas stream with minimal memory effects.

The instrument is simple to use; and liquid flows and plasma conditions are stabilized within a few minutes. The interface vessel provides a simple visual check on instrument performance prior to analysis. Once stable conditions are obtained, the instrument can be operated at a rate of up to 60 samples/h. The computer interface sends pulses to the computer system to measure blank or sample levels by integration.

The Plasma-Therm Hydride Generator rapidly provides reliable precise results with detection levels of 0·1 ppb and, most importantly, it provides a rapid rise to signal maximum and a small memory effect, rapidly reducing to within 1% of the background level. The system can be linked quickly and simply to any ICP or AA system, thus greatly extending the versatility of the basic instrument. The instrument package is competitively priced at £3500.

*More details from Plasma-Therm Ltd, Unit 3, 2/3 Kangley Bridge Road, Lower Sydenham, London SE26 5AR. Tel.: 01 778 6798.*

Circle No. 39 on Reader Enquiry Card

### Johnson Matthey & Malthus Instruments

Johnson Matthey have acquired Malthus Instruments. The Perth-based company have developed a system for measuring the growth of bacteria by monitoring conductivity changes. Malthus's system has a particular value in hospitals, for example by indicating the optimum drug treatment for patients with blood or urinary tract infections, monitoring the progress of transplant operations and the effects of chemotherapy. Malthus's instruments will now be marketed from Matthey's Printed Products subsidiary.

*Further information from Matthey Printed Products Ltd, William Clowes Street, Burslem, Stoke-on-Trent ST6 3AT, UK. Tel.: 0782 85631.*

Circle No. 40 on Reader Enquiry Card

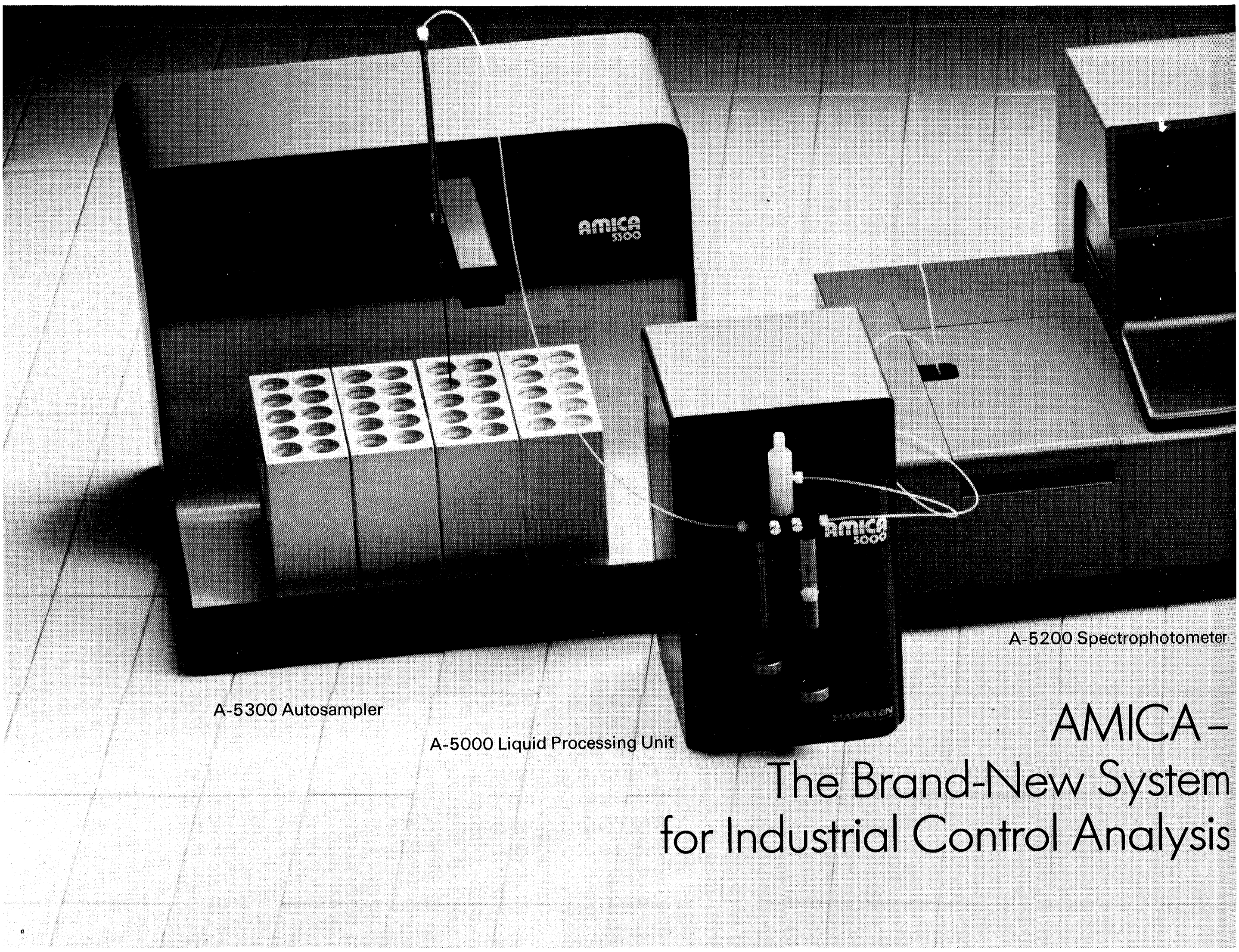

### 'Aspects'

The latest number of *Aspects*—Erba Science (UK) Ltd's occasional newsletter—contains articles on monitoring inert atmospheres, analysis of water pollutants and environmental particulate matter plus information on the company's new products and literature.

*Requests to be included on the free mailing list for the newsletter to Erba Science (UK) Ltd, Headlands Trading Estate, Swindon, Wiltshire SN2 6JQ, UK. Tel.: 0798 33551.*

Circle No. 41 on Reader Enquiry Card

### Temperature controller

The Series 48 is a 96×48 mm plug-in three-term temperature controller, which was announced by Newtronic Controls International Ltd in November. It gives three-term control up to 1599°C and incorporates variable overshoot inhibition which can be adjusted to suit the response of the load being controlled. Once stabilized at the required temperature, the controller will maintain the set point regardless of variations in ambient temperature, control power requirements or mains voltage. A meter is provided calibrated in centigrade. Overall accuracy is said to be 0·25% of span. It accepts inputs from BS or DIN thermocouples or resistance thermometers. Outputs for relays are provided, as are logic signals to drive compatible thyristor stacks up to 400 A rating. High or low alarm levels can be specified and these have front-panel LED indicators.

The controller is likely to be suitable for a variety of applications, but Newtronic Controls International believe that its small size will make it particularly useful for multi-zone control where panel space is at a premium.

*A leaflet is available from Newtronic Controls International Ltd, Stag Industrial Estate, Atlantic Street, Broadheath, Altrincham, Cheshire WA14 5NN, UK. Tel.: 061 928 4275.*

Circle No. 42 on Reader Enquiry Card

### Infra-red spectra handbook

A new handbook, which contains over 500 infra-red spectra of hazardous chemicals, is available from Heyden & Son—it was originally published by Sadtler Research Laboratories. Intended for chemists involved in analysing, monitoring, controlling or studying environmental or occupational pollutants and chemical spills, the book contains spectra indexed alphabetically, by molecular formula, and by strongest band for rapid identification. The spectra are presented on a linear transmittance versus frequency (wavenumber) format over the spectral region 4000 to 400 $cm^{-1}$. The name of the compound, the molecular formula, the structure, the source of sample, the CAS Registry number and the NIOSH number are presented for each compound along with the spectrum. Infra-red vapour-phase spectra for appropriate compounds are presented next to the corresponding infra-red condensed-phase spectra. For these compounds, physical properties and instrumental settings are also presented with the spectrum.

*Orders for 'The Handbook of Priority Pollutants and Toxic Chemicals' to Heyden & Son Ltd, Spectrum House, Hillview Gardens, London NW4 2JQ. Tel.: 01 203 5171.*

Circle No. 43 on Reader Enquiry Card

### Linear analyser for radio TLC

Linear analysis, according to LabLogic of Sheffield, UK, largely replaces scanning TLC plates because it makes a measurement in minutes and achieves better limits

Circle No. 44 on Reader Enquiry Card of detection. The linear analyser they offer uses a position-sensitive proportional gas flow counter which is placed over the whole trace and coupled to a delay line; the result is digitized and read into a computer memory. A growing picture is displayed. Regions of interest can be marked and integrated and data is stored on disc for recall. The measurement table will take up to 4 20 cm × 20 cm TLC plates for automatic processing, the detector being moved from trace to trace. New plates can be added and parameters entered whilst measurement is taking place. Output is either a copy of the VDU screen or a 1 : 1 representation of the TLC plate. To be consistent with GLP, no data is lost and all computations are noted.

*Details from LabLogic, Hallamshire Technical Services Ltd, 72 Eldon Street, Wellington Industrial Estate, Sheffield S1 4GT, UK. Tel.: 0742 755085.*

**Circle No. 45 on Reader Enquiry Card**

### Chemical option for word-processor

A chemical option is available for Exxon Office Systems' 500 series of information processors. The option allows preparation of research reports, drug analyses, patent applications, professional papers and articles in addition to the 500 series' standard word-processing facilities; the option is expected to be of interest to pharmaceutical companies, to universities and to chemical manufacturers.

Basic rings, double and multi-membered fused structures can be prepared and stored in advance and, when required, can be retrieved, edited and modified by adding side chains. In addition, these bonded structures are displayed so that the operator can see what intricate structures and formulae look like before print-out (steroid rings, polymer chains etc.). Chemical symbols as well as Greek letters, are included on the typewriter-based keyboard; each symbol is produced by a single keystroke and shown on the VDU. And a specially designed printwheel and software allows the reproduction of both text and chemical formulae without the need for printwheel changes or a twin-track printer.

*More information from Exxon Office Systems (UK) Ltd, Borax House, Carlisle Place, London SW1 1HT. Tel.: 01 834 9070.*

**Circle No. 46 on Reader Enquiry Card**

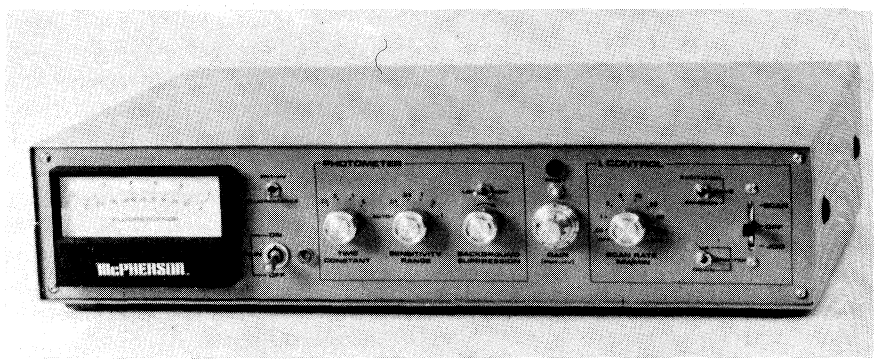

*Schoeffel/McPherson's photometer: the analogue meter output (a digital version is available) and the optional stepper motor controller for grating drive.*

### Photometer and optional stepper controller

For use with the end-on/side-on photomultiplier typically used in optical spectroscopy, Schoeffel/McPherson of Acton, USA, have announced a new photometer with visually indicating autoranging and photomultiplier overload protection/reset control. The unit is described as the missing module between a monochromator/photomultiplier system and its recorder/data interface.

*Literature about the product is available from Schoeffel/McPherson, Acton Facility, 530 Main Street, Acton, Massachusetts 01720, USA. Tel.: 617 263 7733.*

**Circle No. 47 on Reader Enquiry Card**

### UV/COD monitor for water-pollution control

An automatic UV/COD monitor, the Model 101, is able, using a dual UV/Vis absorption technique, to instantly and precisely measure the level of organic compound contamination in water. Reproducibility is claimed to be generally better than 1%, and data directly correlates to approved COD methodology. The monitor operates at a 'dual wavelength-dual path' system, which reduces zero drift associated with source detector aging and dirt accumulation in the optical path. The standard wavelengths of 254 nm in the UV range and 546 nm in the visible range compensate for variances of absorbancy caused by sludges in the sample water and lamp deterioration. The typical range span of 0·8 absorbance corresponds to 90 ppm COD. The monitor includes a self-cleaning cell system. The Model 101 UV/COD Monitor is typically used to control municipal waste-water treatment processes and industrial process waters in the metal, pulp and paper, chemical and petroleum industries.

*More information from Astro Resources Corporation, 100 Park Avenue, League City, Texas 77573, USA. Tel.: 713 332 2484.*

**Circle No. 48 on Reader Enquiry Card**

### Data system for interpreting mass spectrometer data

The VG11-250 data system, which is based on the DEC PDP11 16-bit computer, incorporates a range of design features aimed at simplifying the analytical procedures of the mass spectroscopist. These features include three-dimensional plotting, full colour graphics, multi-tasking, high mass capability, automatic calibration and fast data acquisition up to 250 kHz sampling rate. The new data system is capable of displaying information extracted from the various techniques employed by the modern mass spectrometer—very fast scanning, linked scans, fast atom bombardment, ACE, high mass range etc. A combination of up to six independent displays can be viewed at the same time, so providing valuable assistance during comparison, referencing, identification and analytical work.

*More information from VG Analytical Ltd, Tudor Road, Altrincham, Cheshire WA14 5RZ, UK. Tel.: 061 928 6300.*

**Circle No. 49 on Reader Enquiry Card**

